# Identification of errors in the IEDB using ontologies

**DOI:** 10.1093/database/bay005

**Published:** 2018-02-22

**Authors:** Randi Vita, James A Overton, Bjoern Peters

**Affiliations:** Center for Infectious Disease, La Jolla Institute for Allergy and Immunology, 9420 Athena Circle, La Jolla, CA 92037, USA

## Abstract

The Immune Epitope Database (IEDB) is a free online resource that has manually curated over 18 500 references from the scientific literature. Our database presents experimental data relating to the recognition of immune epitopes by the adaptive immune system in a structured, searchable manner. In order to be consistent and accurate in our data representation across many different journals, authors and curators, we have implemented several quality control measures, such as curation rules, controlled vocabularies and links to external ontologies and other resources. Ontologies and other resources have greatly benefited the IEDB through improved search interfaces, easier curation practices, interoperability between the IEDB and other databases and the identification of errors within our dataset. Here, we will elaborate on how ontology mapping and usage can be used to find and correct errors in a manually curated database.

Database URL: www.iedb.org

## Introduction

The Immune Epitope Database (IEDB) (1) describes experiments using up to 400 fields per assay. This data are manually curated, primarily from the scientific literature, following a set of evolving curation rules and utilizing a peer review process (2). Each experiment entered by our curation team depicts the binding of an adaptive immune receptor (T cell receptor, antibody or major histocompatibility complex) to its epitope. An epitope is the portion of a foreign entity (e.g. a viral protein), self-entity (as in autoimmune disease) or allergen that the immune system recognizes. The binding of the immune receptor to its epitope triggers the immune response that either protects one from disease or causes allergic or autoimmune symptoms.

For some of those >400 fields, from the very beginning, we used values from existing resources, such as the National Center for Biotechnology Information (NCBI) taxonomy (3) for organism taxonomical information and the NCBI GenBank (4) sequence database for protein information. Thus, all organism data in the IEDB have always been described by NCBI taxonomy nomenclature. But for many other data fields, we instead maintained our own list of ‘home-made’ terms. These terms covered fields such as ‘Assay Type’, ‘Cell Type’, ‘MHC Restriction’ and so on and were made available to curators as a flat list. These lists were internally vetted and grew over time, as we encountered new terms in the literature. With the goal of becoming more interoperable with other resources and to further standardize the terms used by the IEDB, we iteratively chose fields to map to outside ontologies. This process was informative about our dataset and greatly improved the IEDB.

## Methods and results

### Assessment

Our approach was to first assess the set of terms used by the curators so far, e.g. how many distinct terms were in use for a given data field, how often each term was utilized, how complex was the nomenclature for that data type and so on. Some lists of terms used by the IEDB are fairly short, such as for the field ‘Evidence Code’ (18 terms), while others, such as ‘Assay Type’ (402 terms), are quite long. Some data fields utilized simple terminology, as with ‘Geolocation’, being simply a list of country names, while ‘MHC Restriction’ has complex nomenclature (i.e. HLA-DQA1*03: 01/DQB1*04: 01) which differs for each species. The number of times any given term was utilized provided weight to how likely that term was to be accurate, needed and correctly used. Infrequently utilized terms were scrutinized and often could not be mapped, proving to be errors. As an example, Supplementary Table S1 shows the list of cells types that were in the IEDB’s ‘home-made’ cell types list and how frequently they were used by curators.

### Ontology mapping

We next sought out external ontologies that could meet our needs. In general, we wanted ontologies that were reliable, accessible and accepted as leaders by the biological community. We prioritized The Open Biological and Biomedical Ontology Foundry Ontologies, as they met these criteria (5). The availability of relevant resources aided our selection of which fields to map first. Table 1 lists which ontologies or resources we selected to represent specific IEDB data fields. For example, to map our internal cell types list, we found that we needed to utilize both the cell ontology (CL) (6) and the cell line ontology (CLO) (7), as shown in detail in Supplementary Table S1. CL was used to describe primary cell types, such as splenocyte and lymphocyte, while CLO was needed to describe laboratory generated cell lines, such as JURKAT cell or P815 cell. Once an appropriate resource was identified, all IEDB terms were manually reviewed and mapped to ontology terms and, if not found, new terms were requested. Supplementary Table S1 shows the ontology mappings for IEDB cell types and indicates which terms were newly requested by the IEDB. Our ontology mapping events often led to the creation of a large number of new terms in some ontologies, such as the ontology for biomedical investigations (OBI) (8) with approximately 300 new terms, but with others, such as Uberon (9), no new term requests were needed. This difference reflects the maturity of the resource and the complexity of terms needed in the IEDB. Our list of tissue types, being the source of T cell or antibody studied in the immune epitope assay, was simple, containing common terms such as blood, spleen and lymph node. But the list of experimental assay types, derived from the entirety of the relevant published literature, was quite large and specialized, reflective of the state of the field of immunology and the large number of experiment types utilized over time.

**Table 1.** Ontology or resource selected for IEDB data fields and numbers of errors identified in the IEDB data set, referring to cases where the data was changed as a result of the initial mappingof IEDB terms to an ontology or resource, creation of an immunology specific view of the ontology or resource (tree pruning), or due to annual review of the data at least one year after adoption of the resource for that data field. For fields not yet mapped, the ontology shown is a suggestion, with the actual ontology used to be determined at the time those mappings begin

		Edits made during		
IEDB data field	Ontology/resource used	Initial mapping	Tree pruning	Annual review
Organism	NCBI Taxonomy (3)	NA	5684	68
Protein	GenBank (4), UniProt (17), PDB (18)	NA	3999	310
MHC restriction	MRO (12)	4397	NA	39
Cell type	CO (6), CLO (7)	329	NA	0
Tissue type	Uberon (9)	0	NA	0
Geolocation	Gazetteer (19)	0	NA	0
Assay type	OBI (8)	4983	NA	838
Disease state	DO (16)	2500	NA	TBD
Laboratory animals	MGD (13), RDG (14)	TBD		
Adjuvants	VO (20)	TBD		
Post-translational modifications	PRO (21)	TBD		
Evidence codes	ECO (22)	TBD		

### Error identification

**Figure 1.**
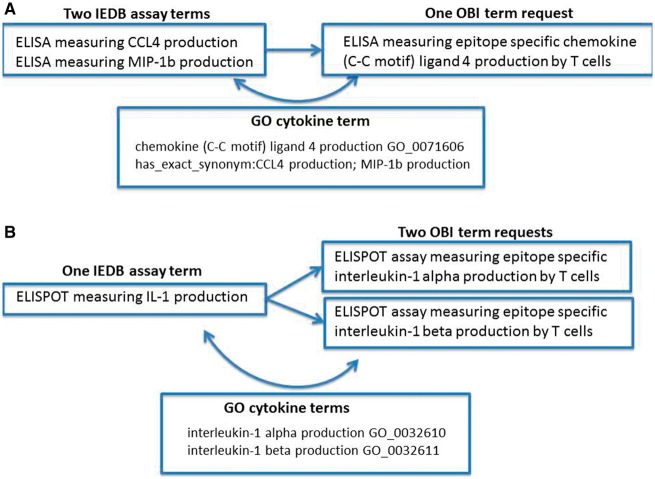
Redundancy and imprecision in IEDB assay type terms. (**A)** Two IEDB terms for the same assay map to a single OBI term request (OBI_0001378). (**B)** One IEDB assay maps to two separate OBI term requests (OBI_0001745 or OBI_0001842).

Error identification and correction were not our original goals of this project; however, during the ontology mapping steps, we identified several error types within our data. We found situations of redundancy in that multiple IEDB terms might map to one ontology term. Figure 1A shows an example of redundancy where two IEDB assay terms (CCL4 and MIP1b) map to a single OBI assay term (CCL4). In this example, we had two assays that measured the same cytokine, but because cytokines use a wide variety of synonyms in the literature that have evolved over time, older manuscripts may refer to the cytokine using one name, while a newer manuscript may use an entirely different name. Because this is a common practice for cytokine terminology and the IEDB had many assay types that measured a wide variety of cytokines, we had several errors of this type in our dataset. It is due to the fact that the OBI assay terms refer to gene ontolgy (GO) (10) biological processes that the redundancy in our assay types was able to be identified. GO curators have compiled extensive synonyms for cytokine terms that allowed us to identify these errors in our flat lists and correct them by mapping all redundant assay terms to a single OBI assay term that refers to the single GO biological process.

We also found imprecision errors where one IEDB term could be mapped to multiple external terms due to the IEDB term being too vague. This error type was also frequently found for assays that measure cytokines due to their nomenclature, as shown in Figure 1B. This example demonstrates where an IEDB assay term, IL-1, was too vague to map to a single OBI assay term, IL-1α or IL-1β. In such cases, we reviewed the original publications and identified the explicit meaning of the authors. These errors typically originated from authors using an abbreviated term for a cytokine in one location of their manuscript, such as IL-1 to mean IL-1α in the figure legend, while using the specific term name in another location, such as in the Materials and Methods section of a published article. As before, these errors were identified by the reference to GO terms by OBI and were corrected as a result of mapping our flat terms list to OBI terms.

### Reasoning

**Figure 2.**
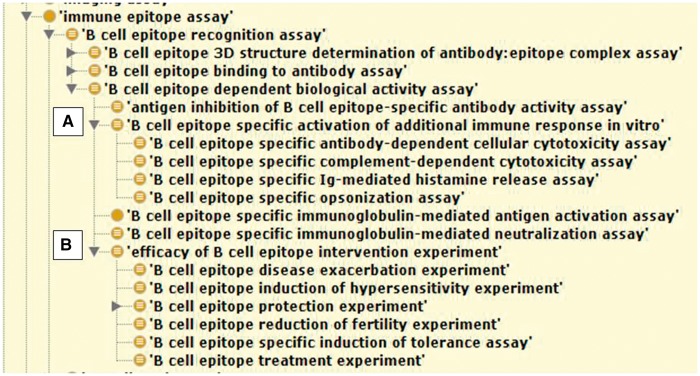
Reasoned OBI hierarchy showing IEDB B cell assays with two distinct assay groups reflective of correlates of protection in (**A)**. and true measures of *in vivo* protection in (**B)**.

Once all IEDB terms for a select data field were mapped to an ontology, we next viewed a reasoned OWL file of our terms in the context of the external ontology. This allowed us to see our data in a whole new way. Now, rather than a flat list of terms, our terms were organized within the hierarchy of the ontology with parents, siblings and children. If terms that we expected to be siblings showed up in very different regions of the tree, this often indicated curation errors, while in other cases, it was a learning opportunity. For example, some assay types might be grouped together by immunologists, including our curators, as demonstrating protection from disease, such as an assay that measures the neutralization of a virus by an antibody and an assay that measures protection from viral challenge when the same antibody is administered to a mouse. However, once reasoned, some assay types, like neutralization, were shown to be measures of downstream processes *in vitro* (correlates of protection) while others, like protection, were shown to be true measures of *in vivo* protection, as shown in Figure 2. Thus, the ontology’s hierarchy taught us about our data, such that we now recognized two distinct sets of assay types, one indicating correlates of protection and the other demonstrating true *in vivo* protection, where we previously thought of them as roughly equivalent siblings. Our curation rules take similarities and differences of term meanings into account, so this information is quite valuable and affects how curators capture data. Therefore, curators will no longer group these dissimilar assay types together.

### Pruning

A related, but separate task that we performed was the creation of immunology specific views of existing hierarchies, as previously published (11). We refer to the creation of these views as ‘tree pruning’. The IEDB always utilized NCBI taxonomy to describe organisms, but this included the entire NCBI taxonomic tree, which contains a very large number of nodes, most of which were irrelevant to the IEDB data and users. Thus, we pruned the NCBI tree to contain only those nodes that we needed in order to represent our data. This process also surprisingly identified curation errors. We had expected that because we had always used a defined hierarchy, errors would be rare. However, as we analyzed how much data utilized each branch and node of the NCBI tree, we found outliers where there were few usages. Many of these were found to be curation errors, where the curator mistakenly chose an incorrect term. Examples included using a more unusual term such as Mus sp. ‘mice’ (NCBI Taonomy ID 10095) instead of the more commonly used *Mus musculus* ‘mouse’ (NCBI Taonomy ID 10090) to describe experiments using mice, which is not a true error, but by having all similar data in the IEDB utilize the exact same NCBI terms, valuable grouping of data from a large number of related publications becomes possible. Similarly, we recurated cases where curators chose more vague terms when more specific terms were available. An example of this is when Human Herpesvirus 6 (NCBI Taonomy ID 10368) was curated, but instead Human betaherpesvirus 6 A (NCBI Taonomy ID 32603) or Human betaherpesvirus 6B 6 (NCBI Taonomy ID 32604) could have been selected.

Additionally, we used the pruned NCBI tree to generate a protein tree that organized reference proteomes for each species into a hierarchy. The process of mapping protein terms utilized by our dataset into this organized tree also identified errors. Similar to the NCBI tree pruning event, outliers with few usages were often identified as curation errors.

Just the process of scrutinizing what terms the IEDB used and how they were being used made us aware of errors in our data and was a valuable endeavor. Each time we adopted an ontology, we gained new information about our data and identified errors that we would not have found otherwise. In all, we found over 20 000 cases where we changed IEDB data as a result of the incorporation of ontologies or other resources. These were related to roughly 125 distinct categories of problems identified (exact data on the number of distinct problems was not tracked). Some cases were true errors and some were opportunities to add more detail than we had previously. Table 1 shows the numbers of edits made to an individual assay per ontology used. The same error may have occurred in many assays and these numbers reflect individual data changes rather than individual error types. Thus, we found the process of mapping flat lists of home-made terms to ontology terms and the further pruning of these ontologies to be a great opportunity to improve our dataset.

We have not yet translated all of our term lists to formal ontologies and plan to continue doing so, as soon as we can identify appropriate ontologies. In some cases, such as with MHC nomenclature, we created a new ontology, the MHC Restriction Ontology (MRO) (12). We did this because an adequate resource did not yet exist and because this area of research overlaps with our area of expertise. However, we do not plan on creating new ontologies every time we want to map one of our data fields, instead, preferring to wait for outside experts. For example, laboratory animal strains are not included in NCBI Taxonomy, so they represent an area currently lacking coverage in the IEDB. The Mouse Genome Database (MGD) (13) and Rat Genome Database (RGD) (14) are experts in laboratory mouse and rat strains, respectively, and we plan on utilizing their identifiers as soon as the majority of the strains referenced by our dataset have formal MGI or RGD identifiers. Other IEDB data fields that we plan to convert to ontology terms include, but are not limited to adjuvants, post-translational modifications and evidence codes. We will determine which ontologies to use for these fields at the time of mapping, however, Table 1 shows potential ontologies for these cases.

### Implementation

Once errors in our data were corrected, we added each ontology tree to the curation and search interfaces as ‘Finder’ applications to present the terms as a hierarchical tree rather than a flat list. This led to more accurate curation and improved search capabilities. For curators, seeing the possible selections in a reasoned tree decreases the number of errors as the hierarchy helps them make better selections. After the adoption of a new ontology as a ‘Finder’ application in the IEDB, we continue to assess its success by performing an annual review every year. These reviews involve determining how often each term was utilized, reviewing the hierarchies for unusual nodes and spot checking the data in depth. The numbers of errors found during the reviews have been quite small and are shown in Table 1 in the ‘Annual Review’ column for the first review performed approximately one year after adoption of each resource. We continue to review each in every subsequent year and so far, each repeated review has found less and fewer errors.

For our end users, the hierarchy allows them to search data in more meaningful ways, e.g. all assays that measure the same cytokine are now grouped together instead of being listed as separate members of a flat list. This allows for searches at various level of detail in the hierarchy, something that was not possible previously.

### Validation

Additionally, the logical definitions were used to generate validation rules to identify additional errors (15). Once data fields were mapped to an ontology, the logical definition of the ontology term can be used to restrict values of related fields. For example, the disease ontology (DO) (16) logically defines certain diseases as being caused by specific pathogens or allergens. The IEDB has a field for the ‘Disease State’ of the host and a separate field for the ‘Immunogen’ that caused the disease. For example, dengue fever (‘Disease State’) is caused by dengue virus (‘Immunogen’). As these are separate fields in the IEDB, curators complete them independently. By mapping the ‘Disease State’ field to the DO term, we can now use the logical definition provided by DO to find errors in the independently curated ‘Immunogen’ field. Thus, for any case where ‘Disease State’ is curated as dengue fever must have the ‘Immunogen’ curated as dengue virus, otherwise it is a curation error that we are now aware of and can correct. Further, automated validation can be built to enforce these relationships. We have future plans to take this concept even further to auto-populate fields based upon ontology logical definitions. This would, e.g. auto-populate the ‘Immunogen’ field as dengue virus every time the curator selects the ‘Disease State’ of dengue fever. This will not only save curator time but will also prevent errors.

## Discussion

Ontologies offer great benefits to manually curated databases. They provide synonyms, textual and logical definitions and hierarchical relationships between terms. Mapping lists of terms from the literature to formal ontology terms standardizes datasets and identifies errors, as it requires close scrutiny of what terms are used and how they are defined. Any effort that requires one to thoroughly review their datasets and curation practices would be expected to identify errors, however, the use of formal ontologies to drive such a process offers a systematic, structured approach and the expertise of each external resource.

Curators also benefit from the expertise of each external ontology, when they view familiar terms in a reasoned hierarchy, resulting in better curation. Subsequent to mapping IEDB terms to formal ontologies, we have performed annual reviews of the IEDB data utilizing each ontology. In these reviews, we have identified far fewer errors, as shown in Table 1. Once an ontology is adopted by the IEDB, if new terms are encountered in the literature, we simply make a new term request to the ontology and refresh our Finders. This process enriches the ontologies we use and helps maintain the quality of our data.

Finally, because our database users are also the authors of the papers that we curate, we are hopeful that their exposure to formal nomenclature via our ontology driven website will improve the terminology that they utilize in their publications. We hope our experiences in finding and correcting errors using ontologies will inspire other projects to do the same.

## Supplementary data

Supplementary data are available at *Database* Online.

## Supplementary Material

Supplementary Table

## References

[1] Vita R., Overton J.A., Greenbaum J.A. (2015) The immune epitope database (IEDB) 3.0. Nucleic Acids Res., 43, D405–D412. 25300482 10.1093/nar/gku938 PMC4384014

[2] Vita R., Peters B., Sette A. (2008) The curation guidelines of the immune epitope database and analysis resource. Cytometry A, 73A, 1066–1070. 10.1002/cyto.a.20585 PMC2597159 18688821

[3] NCBI Resource Coordinators. (2017) Database Resources of the National Center for Biotechnology Information. Nucleic Acids Res., 45, D12–D17. 27899561 10.1093/nar/gkw1071 PMC5210554

[4] Benson D.A., Karsch-Mizrachi I., Lipman D.J. (2011) GenBank. Nucleic Acids Res., 39, D32–D37. 21071399 10.1093/nar/gkq1079 PMC3013681

[5] Smith B., Ashburner M., Rosse C. (2007) The OBO Foundry: coordinated evolution of ontologies to support biomedical data integration. Nat. Biotechnol., 25, 1251–1255. 17989687 10.1038/nbt1346 PMC2814061

[6] Meehan T.F., Masci A.M., Abdulla A. (2011) Logical development of the cell ontology. BMC Bioinformatics, 12, 6. 21208450 10.1186/1471-2105-12-6 PMC3024222

[7] Sarntivijai S., Lin Y., Xiang Z. (2014) CLO: the cell line ontology. J. Biomed. Semantics, 5, 37. 25852852 10.1186/2041-1480-5-37 PMC4387853

[8] Bandrowski A., Brinkman R., Brochhausen M. (2016) The ontology for biomedical investigations. PLoS One, 11, e0154556. 27128319 10.1371/journal.pone.0154556 PMC4851331

[9] Mungall C.J., Torniai C., Gkoutos G.V. (2012) Uberon, an integrative multi-species anatomy ontology. Genome Biol., 31; 13, R5. 22293552 10.1186/gb-2012-13-1-r5 PMC3334586

[10] Ashburner M., Ball C.A., Blake J.A. (2000) Gene ontology: tool for the unification of biology. Nat. Genet., 25, 25–29. The Gene Ontology Consortium. 10802651 10.1038/75556 PMC3037419

[11] Vita R., Overton J.A., Sette A. (2017) Better living through ontologies at the Immune Epitope Database. Database, 2017, 1–7. 10.1093/database/bax014 PMC5467561 28365732

[12] Vita R., Overton J.A., Seymour E. (2016) An ontology for major histocompatibility restriction. J. Biomed Semantics, 7, 1. 26759709 10.1186/s13326-016-0045-5 PMC4709943

[13] Blake J.A., Eppig J.T., Kadin J.A. (2017) Mouse Genome Database (MGD)-2017: community knowledge resource for the laboratory mouse. Nucleic Acids Res., 45, D723–D729. 27899570 10.1093/nar/gkw1040 PMC5210536

[14] Shimoyama M., De Pons J., Hayman G.T. (2015) The Rat Genome Database 2015: genomic, phenotypic and environmental variations and disease. Nucleic Acids Res., 43, D743–D750. 25355511 10.1093/nar/gku1026 PMC4383884

[15] Vita R., Overton J.A., Greenbaum J.A. (2013) Query enhancement through the practical application of ontology: the IEDB and OBI. J. Biomed. Semantics, 4(Suppl 1), S6. 23734660 10.1186/2041-1480-4-S1-S6 PMC3633001

[16] Schriml L.M., Arze C., Nadendla S. (2012) Disease ontology: a backbone for disease semantic integration. Nucleic Acids Res., 40, D940–D946. 22080554 10.1093/nar/gkr972 PMC3245088

[17] The UniProt Consortium. (2017) UniProt: the universal protein knowledgebase. Nucleic Acids Res., 45, D158–D169. 27899622 10.1093/nar/gkw1099 PMC5210571

[18] Berman H.M. (2000) The protein data bank. Nucleic Acids Res., 28, 235–242. 10592235 10.1093/nar/28.1.235 PMC102472

[19] http://www.obofoundry.org/ontology/gaz.html (7 December 2017, date last accessed).

[20] Lin Y., He Y. (2012) Ontology representation and analysis of vaccine formulation and administration and their effects on vaccine immune responses. J. Biomed. Semantics, 3, 17. 23256535 10.1186/2041-1480-3-17 PMC3639077

[21] Natale D.A., Arighi C.N., Blake J.A. (2014) Protein Ontology: a controlled structured network of protein entities. Nucleic Acids Res., 42, D415–D421. 24270789 10.1093/nar/gkt1173 PMC3964965

[22] Chibucos M.C., Mungall C.J., Balakrishnan R. (2014) Standardized description of scientific evidence using the Evidence Ontology (ECO). Database, 2014, 1–11. 10.1093/database/bau075 PMC4105709 25052702

